# An enhanced toolkit for the generation of knockout and marker-free fluorescent *Plasmodium chabaudi*

**DOI:** 10.12688/wellcomeopenres.15587.2

**Published:** 2020-06-24

**Authors:** Edward J Marr, Rachel M Milne, Burcu Anar, Gareth Girling, Frank Schwach, Jason P Mooney, Wiebke Nahrendorf, Philip J Spence, Deirdre Cunningham, David A Baker, Jean Langhorne, Julian C Rayner, Oliver Billker, Ellen S Bushell, Joanne Thompson

**Affiliations:** 1 Institute of Immunology and Infection Research, School of Biological Sciences, University of Edinburgh, Ashworth Laboratories, The King's Buildings, Edinburgh, EH9 3FL, UK; 2 Parasites and Microbes, Wellcome Sanger Institute, Wellcome Genome Campus, Cambridge, CB10 1SA, UK; 3 The Roslin Institute and Royal (Dick) School of Veterinary Studies, University of Edinburgh, Edinburgh, Midlothian, EH25 9RG, UK; 4 The Francis Crick Institute, London, NW1 1AT, UK; 5 Faculty of Infectious and Tropical Diseases, London School of Hygiene and Tropical Medicine, Keppel Street, London, WC1E 7HT, UK; 6 Cambridge Institute for Medical Research, University of Cambridge, The Keith Peters Building, Hills Road, Cambridge, CB2 0XY, UK; 7 Laboratory for Molecular Infection Medicine Sweden, Department of Molecular Biology, Umeå University, Umeå, 901 87, Sweden

**Keywords:** Plasmodium chabaudi, malaria, Transfection, PlasmoGem

## Abstract

The rodent parasite *Plasmodium chabaudi *is an important *in vivo *model of malaria. The ability to produce chronic infections makes it particularly useful for investigating the development of anti- *Plasmodium* immunity, as well as features associated with parasite virulence during both the acute and chronic phases of infection. *P. chabaudi* also undergoes asexual maturation (schizogony) and erythrocyte invasion in culture, so offers an experimentally-amenable *in vivo *to *in vitro *model for studying gene function and drug activity during parasite replication. To extend the usefulness of this model, we have further optimised transfection protocols and plasmids for *P. chabaudi* and generated stable, fluorescent lines that are free from drug-selectable marker genes. These mother-lines show the same infection dynamics as wild-type parasites throughout the lifecycle in mice and mosquitoes; furthermore, their virulence can be increased by serial blood passage and reset by mosquito transmission. We have also adapted the large-insert, linear *Plasmo*GEM vectors that have revolutionised the scale of experimental genetics in another rodent malaria parasite and used these to generate barcoded *P. chabaudi *gene-deletion and –tagging vectors for transfection in our fluorescent *P. chabaudi* mother-lines. This produces a tool-kit of *P. chabaudi *lines, vectors and transfection approaches that will be of broad utility to the research community.

## Introduction

The rodent parasite *Plasmodium chabaudi* is a leading *in vivo* model for the study of host immunity and immunopathology in malaria, and provides an extremely powerful tool to study the parasite biology and parasite-host interactions that underpin virulence, chronicity and transmission ( Spence *et al.*, 2013; Spence *et al.*, 2015). This is because *P. chabaudi* demonstrates sequestration and rosetting at the mature schizont stage and resolves to establish a chronic infection with periodic recrudescence and antigenic variation, in a similar manner to the dominant cause of human malaria mortality, *P. falciparum* (reviewed in ( Langhorne *et al.*, 2002; Stevenson & Riley, 2004)). Furthermore, commitment to sexual development (gametocytogenesis) increases *after* the resolution of acute infection, and development in the mosquito progresses through each bottleneck with physiologically relevant parasite numbers ( Spence *et al.*, 2012). In addition, there are a large number of phenotypically diverse parasite genotypes available, which provide a unique opportunity to a) develop co-infection, superinfection and re-infection models and b) interrogate the role of parasite genetics in shaping the course and outcome of infection. *Plasmodium chabaudi* is therefore an extremely adaptable model that can be used to study every step in the *Plasmodium* life cycle.

Despite the phenotypic advantages of this model system, the development of transfection technologies for *P. chabaudi* has lagged behind those for other rodent malaria models. Methods for generating *Plasmodium chabaudi* reporter lines by single cross-over recombination and genomic integration of a fluorescent or luminescent protein cassette have, however, been described ( Brugat *et al.*, 2014; Reece & Thompson 2008; Spence *et al.*, 2011), and these parasites and transfection methods have proved useful for imaging parasite accumulation and associated pathology in host tissues ( Brugat *et al.*, 2014; Borges da Silva *et al.*, 2015; Brugat *et al.*, 2017; Cunningham *et al.*, 2017; Medeiros *et al.*, 2013), as well as for visualising parasite protein localisation ( Yam *et al.*, 2016). Nevertheless, the relative inefficiency of these techniques has hindered the progress of *P. chabaudi* experimental genetic studies.

To improve the accessibility and reproducibility of *P. chabaudi* transfection techniques, we have optimised methods that enable parasite purification and transfection in the laboratory and drug selection in wild-type mice, and have used these methods to generate stable, highly-fluorescent lines by double cross-over integration of genes encoding green- and mCherry-fluorescent proteins ( Shaner *et al.*, 2004) into a dispensable gene locus, using a transfection vector that allows recycling of the drug-selectable marker. These parasites are viable throughout the lifecycle in mice and mosquitoes and can be used as a fluorescence mother-line. We tested this by adapting high efficiency large-insert *Plasmo*GEM vectors and used them to generate gene-modified fluorescent parasite lines ( Pfander *et al.*, 2011). Together, this toolkit provides a clear way forward for future expansion of gene targeting studies in *P. chabaudi.*

## Methods

### Ethical statement and mouse procedures at the University of Edinburgh

All procedures were carried out in accordance with the UK Home Office regulations (Animals Scientific Procedures Act, 1986; project licence number 70/8546 and P04ABDCAA) and approved by Ethical Review Body of the University of Edinburgh. Throughout this study, all efforts were made to reduce animal usage and ameliorate harm to animals. Mice were housed in the University of Edinburgh Licenced Animal Facilities 60/2605), and all animal procedures were performed in laboratories within the animal facilities. C57Bl/6Jax mice were bred under specific pathogen free conditions at the University of Edinburgh. Experimental female C57Bl/6 mice, aged six to twelve weeks, were kept in specific-pathogen-free conditions and subjected to regular pathogen monitoring by sentinel screening. Mice were housed with at least one companion in individually ventilated cages furnished with autoclaved woodchip, fun tunnel and tissue paper at 21 ± 2°C under a reverse light-dark cycle (light, 19.00 – 07.00; dark, 07.00 – 19.00) at a relative humidity of 55 ± 10%. Mice were housed under these light-dark cycle conditions to allow collection of *P. chabaudi* trophozoites prior to schizogony at 13.00-15.00 hrs, and were allowed to adapt to a reverse-light schedule for at least 7 days before *P. chabaudi* infection. They were fed a commercially available, autoclaved dry rodent diet (Rat and Mouse No. 3 Breeding diet; Special Diets Services) and water, both available *ad libitum*. The health of mice was monitored by routine daily visual health checks, and *P. chabaudi*-infected mice were monitored at least twice daily.

### Mice, mosquitoes and parasites

The C57Bl/6 mouse *Plasmodium chabaudi chabaudi* AS animal model of malaria was chosen to minimize host genetic variability and to obtain robust infections with a very low incidence of severe disease. *P.c. chabaudi* AS parasites were obtained from the European Malaria Reagent Repository at the University of Edinburgh. *Anopheles stephensi* were maintained, and transmission of *P. chabaudi* was carried out, as described in ( Spence *et al.*, 2012; Spence *et al.*, 2013). All experimental groups consisted of 2-6 mice housed together. Transfection experiments required 10 mice; two mice to provide infected blood for transfection and eight mice as recipients of parasites transfected with four plasmids in replicate. Parasite cloning required six mice and phenotypic experiments were carried out using groups of 7-8 mice, to provide statistical significance. Euthanasia was performed by cervical dislocation at the end of phenotypic experiments, or by exsanguination under anaesthesia (pentobarbital sodium; Euthatal). Mice were exsanguinated to obtain infected and uninfected blood for transfection experiments, or to collect genetically-modified parasites for storage as stabilates in liquid N _2_. This specific method of anaesthesia reduces animal suffering whilst maximising blood volume obtained.

### Monitoring of infection

Female C57Bl/6Jax mice, aged 6–10 weeks with median weight of 22.5g (IQR 21.7g-23.1g), were inoculated *ip* with 1×10 ^5^ PcAS-GFP _ML_-infected red blood cells and monitored for parasitaemia, weight loss and anaemia daily. Parasitaemia was enumerated either on giemsa-stained, thin-blood smears or by flow cytometric analysis (described in *Underlying data*; Thompson, 2020). In the latter case, 2µL of blood was diluted in 1mL of KSG-H (0.1 M NaCl, 4.6 mM KCl, 1.2 mM MgSO4·7H2, 25 mM Na _2_ HPO _4_, 0.2% (wt/vol) glucose, 25 IU ml ^-1^ heparin sodium), made as described in ( Spence *et al.*, 2011), maintained at 4°C and then diluted a further 1:5 prior to acquisition (150,000 events per sample) on a BD Fortessa analyser (Becton Dickinson, UK) within 2 hours of sampling. Using FlowJo analysis software (Becton Dickinson, UK), gating was performed on singlets (FSC-A v FSC-W), cells (FSC-A v SSC-A) and native GFP (B530/30nm). Free Flow software, FCSalyzer, is available at https://sourceforge.net/projects/fcsalyzer/. GFP gates were set using uninfected blood, with a median of 0.06% background signal and a limit of detection threshold of 0.85% (n=42). For anaemia, haemoglobin (g/L) was determined by Hemocue Hb201+ analyser (Radiometer, Sweden) with moderate anaemia defined as 80-120 g/L and severe anaemia as <80 g/L, in accordance with WHO guidelines. The mean haemoglobin concentration in 36 uninfected control mice was 140 g/L (data was collected from mice at the point when they were assigned to experimental groups in this study).

### Generation of circular transfection vectors

pCAT-230p-G6 was generated by replacing the sil6 target regions of pBAT-SIL6-G6 ( Kooij *et al.*, 2012) with bps 640-1680 and 3312-4255 of the *P. chaubaudi 230p* (PCHAS_0308200) locus. The 230p target regions were amplified from *P.c. chabaudi* AS genomic DNA using primers 5’230pF x 5’230p (5’ target region) and 3’230pF x 3’230pR (3’ target region) and cloned into the multiple cloning sites of pBAT-Sil6-G6 or –M6 to generate pCAT-230p-G6 or pCAT-230p–M6. Plasmids to delete PCHAS_ 0812700 (PcCRMP1) were generated by replacing the sil6 target regions of pBAT-SIL6-M6 with bp 2-778 (5’) and 6855-7849 (3’) of *pccrmp1*.

### Construction and mapping of *Plasmo*GEM *P. chabaudi* genomic clone libraries

*Plasmo*GEM, the *Plasmodium* genetic modification project, is a collaborative project at the Wellcome Sanger Institute with the goal of developing, distributing and applying large-scale resources for *Plasmodium* genetic modification.

Blood stage *P. chabaudi* AS parasites were purified using Plasmodipur (EuroProxima) and MACS Columns (Miltenyi Biotec). Parasite genomic DNA was purified using the Qiagen DNeasy Blood & Tissue Kit (catalogue # 69504). Nuclear DNA was isolated from the preparation (removing the 6 kb mitochondrial genome) by gel electrophoresis and purification of the high molecular weight nuclear genome band. Libraries of genomic DNA inserts were generated using the BigEasy v2.0 Linear Cloning System (pJAZZ-OK Blunt Vector, Lucigen, USA; catalogue # 43036-1) as described ( Pfander *et al.*, 2011). Briefly; 10 µg genomic DNA was mechanically sheared and end-repaired using mung bean nuclease prior to ethanol precipitation. DNA was pelleted and resuspended in TE buffer, then size selected on a 0.8% agarose gel. DNA fragments of 6-8, 8-10 and 10-15 kb were excised, purified and end-repaired using the Lucigen kit. After an additional gel size selection, each insert size range was ligated individually into the pJAZZ vector. The duplicated end-repair and size-selection steps improves cloning efficiency and accuracy of size selection. Ligation reactions were purified using phenol:chloroform extraction and ethanol precipitation, and resuspended in dH _2_O prior to electroporation into BigEasy TSA cells (Lucigen, USA, catalogue # 60224-1). TSA cells harboring *P. chabaudi* genomic library clones within pJAZZ vectors were propagated in Terrific Broth (TB) medium supplemented with 0.4% glycerol and 30 µg/ml kanamycin as recommended by the manufacturer, without arabinose induction.

*Plasmodium chabaudi* genomic library (PcG01 and PcG02) clones were arrayed on 96-well plates and subjected to capillary sequencing of the gDNA ends. To locate each gDNA insert, each library clone was mapped to version 3 of the *P. chabaudi* (AS strain) genome ( Otto *et al.*, 2014), using SMALT (version 0.7.5) as described previously ( Pfander *et al.*, 2011).

### Design and generation of *Plasmo*GEM gene targeting vectors for *P. chabaudi*

We created designs for all possible gene knock-out and C-terminal tagging vectors from the mapped PcG01 and PcG02 library using a custom software pipeline as previously described for generating a *P. berghei* resource ( Schwach *et al.*, 2015).

Plasmids and protocols for the parallel recombinase-mediated engineering (recombineering) of PcG01 and PcG02 library clones on 96-well plates were the same as used previously for the generation of *P. berghei* vectors ( Pfander *et al.*, 2011). Briefly, (1) TSA cells harboring PcG01 and PcG02 library clones were made transiently recombineering-competent by introduction of the pSC101gbdA plasmid that encodes elements of the *red*/ET operon of bacteriophage lambda ( Zhang *et al.*, 1998). (2) The target genes encoded by the PcG01 and PcG02 library clones were subsequently modified by site-specific recombineering mediated by 50 bp homologous targeting regions so to delete or disrupt the open reading frame (knock-out vectors), or delete the stop codon (tagging-vectors). The recombineering step introduces a dual (zeo-pheS) bacterial selectable marker which confers resistance to zeocin and sensitivity to 4-chloro-DL-phenylalanine. (3) In a LR-Gateway Clonase I (Thermofisher, catalogue # 11791042) mediated reaction with the pR6K attL1-3xHA-hdhfr/yfcu-attL2 plasmid, which also introduces a 3xHA epitope tag (silent in KO parasites), the zeo-pheS selection cassette was then replaced by a mutated version of human dihydrofolate reductase ( *hdhfr*) coupled to uridyl phosphoribosyl transferase (yFCU), which confers resistance to pyrimethamine and sensitivity to 5-fluorocytosine (5-FC) in *Plasmodium*. The *hdhfr*-yFCU cassette in pR6K attL1-3xHA-hdhfr-yfcu-attL2 ( Pfander *et al.,* 2011) is flanked by duplicated *pbdhfr* 3’UTR sequences, facilitating efficient recombination under 5-FC pressure.

Final vectors were subjected to full-length Illumina sequencing on a MiSeq platform to confirm the correct sequence of the homology arms before uploading design data to our public resource at https://plasmogem.sanger.ac.uk ( Schwach *et al.*, 2015).

SC101gbdA was propagated at 30°C in DH10B cells in 2x low salt Luria Broth (LB) with 5 µg/ml tetracycline, or together with pJazz library clones in TB medium with 0.4% glycerol + 30 µg/ml kanamycin + 5 µg/ml tetracycline in TSA cells. The SC101gbdA and pR6K attR1-zeo ^R^-Phe ^S^-attR2 plasmid served as template for the amplification of the zeo-PheS cassette for the recombineering step. These plasmids were kind gifts from Francis Stewart, Dresden. The Gateway donor plasmid pR6K attL1-3xHA-hdhfr/yfcu-attL2 was the same as used for *P. berghei Plasmo*GEM vectors ( Pfander *et al.*, 2011). The R6K origin of replication plasmids were propagated in PIR1 (Invitrogen) bacteria in LB Broth with 5 µg/ml tetracycline.

### Transfection

For transfection, plasmid DNA was prepared using Qiagen plasmid midi-kit (catalogue # 12143). Circular vectors were linearised with restriction enzymes AatII and ApaLI (New England Biolabs) and purified away from the plasmid backbone using gel electrophoresis and a Qiagen QIAquick gel purification kit (catalogue # 28704), according to the manufacturer’s instructions. DNA was resuspended in dH _2_O at 0.2–1 μg/μl. *The Plasmo*GEM transfection vectors used to delete *pccrmp1* (PCHAS_0812700), *pccrmp2* (PCHAS_ 0617600), *pccrmp3* (PCHAS_0608500), *pccrmp4* (PCHAS_1304000) and PCHAS_0418000 were PGEM-600068, PGEM-597076, PGEM-596604, PGEM-610764 and PGEM-594684, respectively. The *Plasmo*GEM vectors were propagated in *E. coli* TSA cells (Lucigen, USA) according to *Plasmo*GEM protocols ( Pfander *et al.*, 2011; Pfander *et al.*, 2013) and inserts were released by NotI digestion, followed by standard ethanol precipitation.

### *P. chabaudi* schizont culture

The culture of *P. chabaudi* trophozoites to mature schizonts was carried out exactly as described in ( Spence *et al.*, 2011) with the following modifications. To provide parasites for culture, a single wild-type donor C57Bl/6Jax mouse (female, 6–10 weeks) was injected *ip* with 1-5 x 10 ^7^ parasites. At day 3 of infection (parasitaemia 19–45%), the mouse was exsanguinated at 12.00 hrs, and the infected red cells were cultured at 37°C under 5% O _2_, 5% CO _2_, 90% N _2_ in 10 ml complete-RPMI (RPMI 1640 (Invitrogen), 10% (vol/vol) FBS (PAA Laboratories), 6 mM HEPES, 2 mM l-glutamine, 0.5 mM sodium pyruvate and 50 μM β-mercaptoethanol). After 2 hours of culture, flasks were carefully removed from the incubator without disturbing the sedimented cells and maintained at 37°C by placing onto a pre-warmed heat-block or flask. Five ml of culture supernatant was aspirated and replaced with complete-RPMI containing 2 μl of 10 mM compound 2 (C2) Collins *et al.*, 2013). Flasks were re-gassed and returned to the 37°C incubator for a further 2.5 h. C2 was later replaced with ML10, a trisubtituted thiazole *Plasmodium* cGMP-dependent protein kinase (PKG) inhibitor ( Tsagris *et al.*, 2018), which was found to reversibly inhibit the egress of mature schizonts in culture comparably to C2 at concentrations of 0.1-1 μM. ML10 is available from LifeArc.

### Transfection and selection of fluorescent *P. chabaudi* parasites

After 4 hours of culture (30 minutes before the end) a single uninfected wild-type C57Bl/6Jax mouse (female, 6–10 weeks) was exsanguinated to provide fresh red blood cells for invasion by merozoites after electroporation. This uninfected blood was suspended in 10 ml of complete-RPMI and cells were pelleted by centrifugation at 400g for 5 min. The pellet was then resuspended in complete-RPMI to a final volume of 800 μl, and 100 μl aliquots were transferred into 1.5 ml tubes maintained in a heat-block at 37°C.

After 4.5 hours of culture (now enriched in mature arrested schizonts), flasks were carefully removed from the incubator without disturbing the sedimented cells and up to 9 ml of culture supernatant was removed by aspiration and replaced with 10 ml complete-RPMI. Cells were resuspended by gently shaking the flask and 1ml aliquots were transferred to 1.5 ml tubes maintained in a water-bath at 37°C. Cells were pelleted by centrifugation for 20 sec at 2000 rpm in a microcentrifuge and 5 μl of packed cells were transferred to a tube containing 1–5 μg of linearized plasmid in 5–10 μl dH _2_O with 100 μl basic parasite Nucleofector II solution. Cells were gently resuspended, transferred to a cuvette and electroporated in a Nucleofector II (Lonza) using program U-033. Immediately following electroporation, cells were transferred to the tubes containing 100 μl aliquots of fresh red blood cells and maintained at 37°C until all transfections were completed. Each tube of transfected parasites was then injected *iv* into a single wild-type recipient C57Bl/6Jax mouse (female, 6–10 weeks) as described ( Spence *et al.*, 2011). From 24 hours after transfection, recipient mice were supplied with acidified drinking water supplemented with pyrimethamine (35 μg/ml), refreshed every day for 5–12 days until parasites were visible. Mice were exsanguinated by cardiac puncture under terminal anaesthesia to collect infected blood. Correct integration of the resulting PcAS-GFP.Δ230p and PcAS-mCh.Δ230p parasites (and deletion of *230p*) was confirmed by diagnostic PCR using Phusion Blood Direct PCR kit (ThermoFisher Scientific; catalogue # 15280114). All PCR reactions were carried out using an MJ Research DNA Engine, PTC-200. 50 μl reaction mixes contained 25 μl 2x Phusion Blood PCR buffer, 1 μl (0.5 μM) Primers F and R, 1 μl Phusion Blood DNA polymerase, 1 μl whole blood, 22 μl dH _2_O. The cycling protocol was: one cycle x 98°C, 5 min; 40 cycles x 98°C, 1 sec, 72°C (anneal and extend), 30sec; one cycle x 72°C, 1 min.

### Recycling of the drug selection cassettes

Following confirmation of plasmid integration into the *230p* locus, 1 x 10 ^5^ PcAS-GFP.Δ230p parasites were injected *ip* into four wild-type C57Bl/6Jax mice. After 24 h (one cycle of replication), mice were supplied with the pro-drug 5-fluorocytosine in the drinking water (0.5 mg/ml) for 5 days until the parasitaemia of the recycled parasites reached 0.15%. Mice were exsanguinated by cardiac puncture under terminal anaesthesia to collect infected blood.

**Figure 1.**
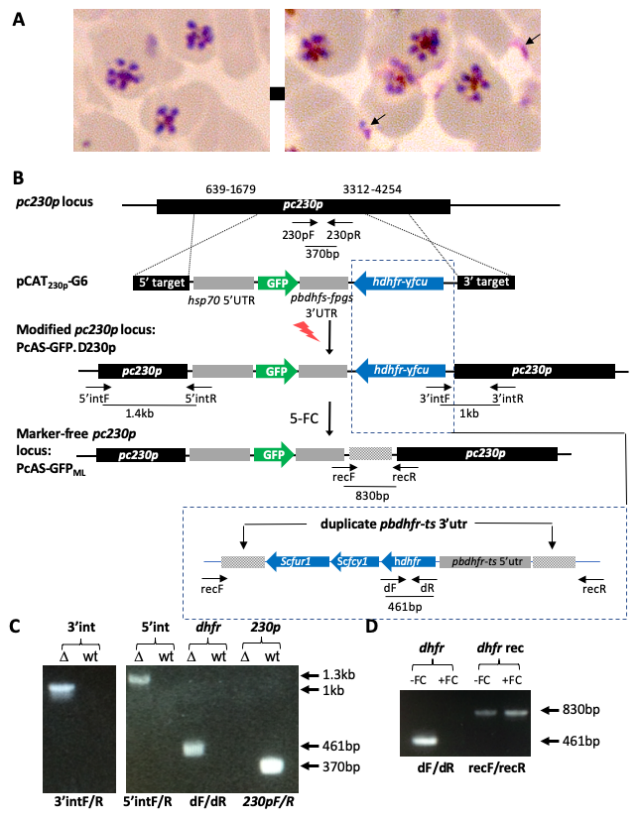
Transfection of *P. chabaudi* AS to create PcAS-GFP _ML_. **A**) Mature *P. chabaudi* AS schizonts accumulate after culture in the presence of C2 (giemsa-stained smear); infected erythrocytes were smeared 5 minutes after C2 was removed from culture. Merozoites undergoing erythrocyte invasion are arrowed. **B**) Schematic representation of the pCAT _230p_-G6 plasmid showing hDHFR and yFcu selectable cassettes, the GFP cassette and 3’PbDHFR-TS direct repeats, allowing recombination and excision of the drug-selectable cassette. **C**) Stable integration of GFP into the *pc230p* locus. To verify correct integration into the *pc230p* locus in PcAS-GFP.Δ230p DNA, 1.4kb and 1kb fragments were amplified from the 5’ and 3’ integration sites using primers 5’intF x 5’intR and 3’intF x 3’intR, respectively. To verify deletion of *pc230p*, primers 230pF x 230pR were used to amplify a 0.37kb fragment in wild-type but not PcAS-GFP.Δ230p DNA. To verify the presence of hDHFR, primers dF1 x dF2 were used to amplify a 461bp fragment in PcAS-GFP.Δ230p but not in wild-type or PcAS-GFP _ML_ DNA. **D**) Recycling of the drug-selectable cassettes. Following selection with 5-FC, recombined parasites excised the drug-selectable cassettes (insert) and an 830bp fragment was amplified from PcAS-GFP _ML_ DNA using primers recF x recR.

Recombination-mediated removal of the drug selectable cassette was determined by diagnostic PCR as described above (Phusion Blood Direct PCR Kit) and the ratio of recombined (primers recF x recR amplicons) to non-recombined parasites (dF x dR amplicons) was compared after 20, 25 and 30 cycles of PCR ( Figure 1D). PcAS-GFP _ML_ parasites showing a ratio > 2:1 recombined:non-recombined parasites were cloned by limiting dilution. Complete recombination and removal of hDHFR was then confirmed by diagnostic PCR as described above using primers recF x recR and dFxdR. A full list of primers used are provided in Table 1.

**Table 1.** Primers. Restriction enzyme sites are underlined.

5’230pF 5’230pR 3’230pF 3’230pR 5’intF (230p) 5’intR (230p) 3’intF (230p) 3’intR (230p) 230pF 230pR dF dR recF recR crmp1F crmp1R crmp1F2 crmp1R2 5’intF (crmp1) 5’intR (crmp1) 3’intF (crmp1) 3’intR (crmp1) GW1 GT1 (crmp1) GT1 (crmp4) Crmp4F Crmp4R	atatt GCCGGCTCATGTTATAACTAGACCATCG ttatt ACTAGTGTACTACCATCTGTTACAC atatt CTCGAGGAATTCTCTTGAACCCGTGGATG ttatt GACGTCGTATGGAACTACATCTATGTAGGAAACTTG GTTATAATTTGTTCGAAACCCTC TAGTATGCACCCTTTGAGGGC AGTAAGAAAAAACGCGTGG CAGTAAAATCACAAACATAAGTATCG GAAAATCCAGAATATGCGTTAGC GACAATGTAATGCTACATATTCTAACG CACCTGGGTATTCTGGC TCAGAACATGGGCATCG GGCCAATTAAAGATAACATCAACATTGAT TTGGACATTTAACTTGAACC ACATATTCACGCCAGGAC TATTCCGCGGATGACAGCTTGAGACTTCTG TCGTGCCCTTTAAATGCACACA AGCTCTCTTTGTATCCTCATGCA TGATGTATTCCCCCCTTCTC TAGTATGCACCCTTTGAGGGC AGTAAGAAAAAACGCGTGG TGGAAATATTGGGTATTGGC CTATTCATACTAGCCATTTTATGTGTG ATATGAAGTGGATGTATTTCGC AAGTAAAAGTCCCATCTGGC TGCATGCACCTGAGTGAATT ACTTTGAAGCAATCGCACAA

## Results

### Design of transfection vectors

P230p has been reported to be dispensable for asexual parasite replication in *P. falciparum*, *P. berghei*, *P. yoelii* and *P. knowlesii* ( Hart *et al.*, 2014; Moon *et al.*, 2013; Thomas *et al.*, 2016; van Dijk *et al.*, 2010). To generate parasites that can be used as fluorescent reference lines throughout the *P. chabaudi* lifecycle, we modified the pBAT-SIL6 plasmid set ( Kooij *et al.*, 2012) that contain cassettes encoding a pyrimethamine-selectable marker (hDHFR) to enable positive selection for transfectants, as well as negative selectable markers yeast cytosine deaminase and uridyl phosphoribosyl transferase (yFCU; confers sensitivity to 5-fluorocytosine (5-FC)) flanked by *pbdhfr-ts* 3’utr duplicate sequences, which promote recombination and recycling of both drug-selectable markers after negative selection with 5-FC ( Figure 1B). Plasmids pCAT _230p_-G6 and pCAT _230p_-M6 are designed to recombine within the *P. chabaudi 230p* locus and introduce genes encoding green or mCherry fluorescent proteins, expressed under the control of the strong and constitutive *P. berghei* HSP70 5’ UTR promoter.

### Generation of stable, marker-free fluorescent *P. chabaudi* mother-lines

Transfection in rodent malaria parasites relies on electroporation of merozoites (the extracellular stage of the blood cycle), which become mechanically released from arrested schizonts during the isolation procedure, and then subsequently re-invade host reticulocytes or erythrocytes after injection into the bloodstream of a recipient mouse. It is likely that transfection efficiency is lower in *P. chabaudi* than in other rodent malaria models because schizont rupture and re-invasion of mature erythrocytes occurs spontaneously in culture, hindering the collection of large populations of arrested schizonts. Therefore, to improve the efficiency of *P. chabaudi* transfection, we tested whether the reversible inhibitor of the *Plasmodium* PKG - C2 ( Collins *et al.*, 2013) - could cause schizonts to arrest and accumulate in culture, thereby increasing the number of viable merozoites available for electroporation.

Addition of C2 at a concentration of 2 μM to the parasite culture led to the accumulation of > 55% mature schizonts ( Figure 1A). Removing C2 (by simply replacing the C2-supplemented media with fresh culture media) allowed normal schizogony to resume and these enriched schizonts could then be electroporated in the presence of plasmids pCAT _230p_-G6 or pCAT _230p_-M6 ( Figure 1B). Next, electroporated merozoites were transferred into tubes containing uninfected mouse erythrocytes held at 37°C to provide an immediate high-density source of fresh red cells for invasion and ring-formation. This step buys enough time (at least 45 minutes) for multiple transfection procedures to be carried out in the laboratory before the transfectants need to be injected into recipient mice. Significantly, these two protocol modifications improved the efficiency of transfection to such an extent that merozoites could be injected directly into wild-type mice, removing the need for immunodeficient mouse strains for parasite expansion and drug selection. In these ways, we could improve the efficiency, practicability and accessibility of this technique. In our laboratory, we have successfully achieved transformation using pCAT-modified vectors targeting six non-essential loci, with transformed parasites becoming patent at days 9-14 after transfection.

To generate stable, marker-free fluorescent *P. chabaudi* mother-lines, plasmids pCAT _230p_-G6 and pCAT _230p_-M6 were transfected into *P. chabaudi* AS parasites according to this modified protocol to introduce GFP and mCherry into the *P. chabaudi 230p* silent locus by double cross-over recombination. After pyrimethamine selection of PcAS-GFP.Δ230p and PcAS-mCh.Δ230p parasites and genotypic verification ( Figure 1C), PcAS-GFP.Δ230p–infected mice were provided with 5-FC to select parasites that had lost the drug-selectable cassettes by homologous recombination of pbdhfr-ts 3’utr duplicate sequences. The resulting marker-free fluorescent parasites were cloned by limiting dilution to produce the mother-line, PcAS-GFP _ML_.

### Fluorescent PcAS parasites transmit through the mosquito and show wild-type infection dynamics

**Figure 2.**
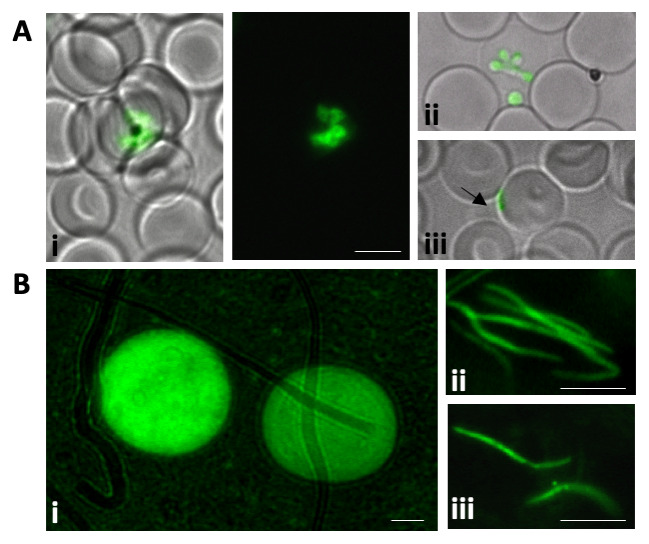
PcAS-GFP _ML_ undergoes schizogony, rupture and rosetting with uninfected erythrocytes in culture and transmit through the mosquito. **A**) PcAS-GFP _ML_ trophozoites from infected mice were cultured for 4.5 hours in the presence of C2. At 4.5hr and in the presence of C2; **i**) schizont-infected erythrocytes form rosettes with uninfected erythrocytes; left–hand panel, merged bright and fluorescent fields of live parasites in culture; right-hand panel, fluorescent field. At 4.5hr and after removal of C2; **ii**) merozoites egress from schizonts and **iii**) invade erythrocytes (arrowed); merged bright and fluorescent fields. Scale bar = 5μm. **B**) **i**) oocysts in the midgut (day 8), Scale bar = 10μm **ii**) midgut sporozoites and **iii**) salivary gland sporozites (day 14). Scale bar = 5μm.

**Figure 3.**
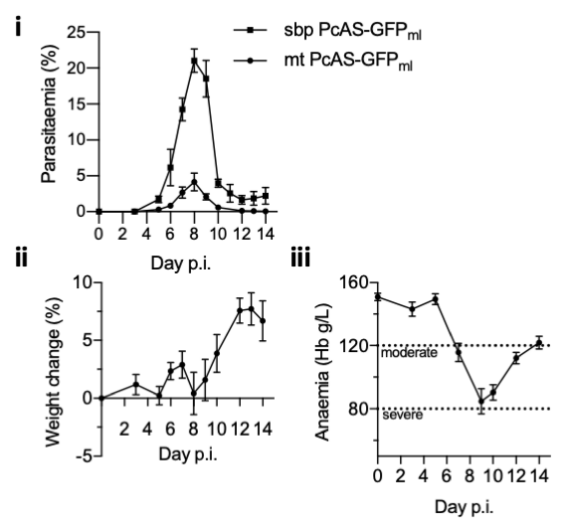
The virulence of PcAS-GFP _ml_ parasites is reset by mosquito transmission and increases after serial passage. C57Bl/6Jax mice were inoculated *ip* with 1x10 ^5^ infected red blood cells of PcAS-GFP _ml_, two blood passages from a mosquito-transmitted inoculum (mt- PcAS-GFP _ml_) or with 1x10 ^5^ infected red blood cells of PcAS-GFP _ml_ that had been serially blood-passaged (sbp PcAS-GFP _ml_). Data shown as a pool of n=7 mice for mt PcAS-GFP _ml_ and n=8 mice for sbp PcAS-GFP _ml_, with mean ± SEM. **i**) Parasitaemia from tail blood, determined by Giemsa stain and by flow cytometric analysis. **ii**) Percent weight change from day 0 post infection (p.i.) with mt PcAS-GFP _ml_. **iii**) Anaemia from day 0 post infection (p.i.) with mt PcAS-GFP _ML_ defined using circulating haemoglobin (Hb g/L) levels, determined by tail blood with a Hemocue analyser. Moderate anaemia is defined as 120 g/L and severe anaemia defined as 80 g/L (dotted lines).

PcAS-GFP _ML_ parasites express GFP strongly throughout the lifecycle and, as with wild-type *P. chabaudi* -AS, undergo schizogony ( Figure 2Aii) and re-invasion of erythrocytes in static culture ( Figure 2Aiii). PcAS-GFP _ML_ schizont-infected erythrocytes also display wild-type cytoadhesion properties by forming rosettes with uninfected erythrocytes ( Figure 2Ai) and PcAS-GFP _ML_ parasites efficiently transmit through the mosquito, giving rise to oocysts ( Figure 2Bi) and salivary gland sporozoites ( Figure 2Biii). Moreover, they establish acute and chronic infections with the same dynamics as wild-type parasites ( Spence *et al.*, 2013), and demonstrate attenuated growth after mosquito transmission ( Figure 3i). As shown previously ( Spence *et al.*, 2013), mice infected with mosquito-transmitted parasites continue to gain weight ( Figure 3ii), but experience significant anaemia that cannot be directly attributed to the destruction of infected erythrocytes ( Figure 3iii). PcAS-GFP _ML_ parasites can therefore be used to investigate every step of the *Plasmodium* life cycle.

### *Plasmo*GEM resources for *P. chabaudi*

We have previously generated large gene deletion and tagging vector libraries for modification of the *P. berghei* genome ( Gomes *et al.*, 2015). Scalability relies on robust, parallel engineering of vectors in 96-well plates using the *red*/ET recombinase system, which in turn requires an arrayed gDNA library, which we constructed in a phage N15-derived linear cloning system with hairpin telomers ( Pfander *et al.*, 2011; Zhang *et al.*, 1998). The low copy number of the pJAZZ cloning vector facilitates serial recombineering, and due to its linear nature it can maintain relatively large AT-rich gDNA fragments ( Godiska *et al.*, 2010), resulting in targeting vectors with large homology regions and high integration rates ( Pfander *et al.*, 2011). Furthermore, since pJAZZ plasmids in our hands are not maintained as episomes when transfected into *P. berghei*, the background noise in transfection experiments is much reduced, such that within a few days of transfection, barcode counting on a sequencer can be used to phenotype dozens of mutants simultaneously ( Bushell *et al.*, 2017; Gomes *et al.*, 2015).

**Figure 4.**
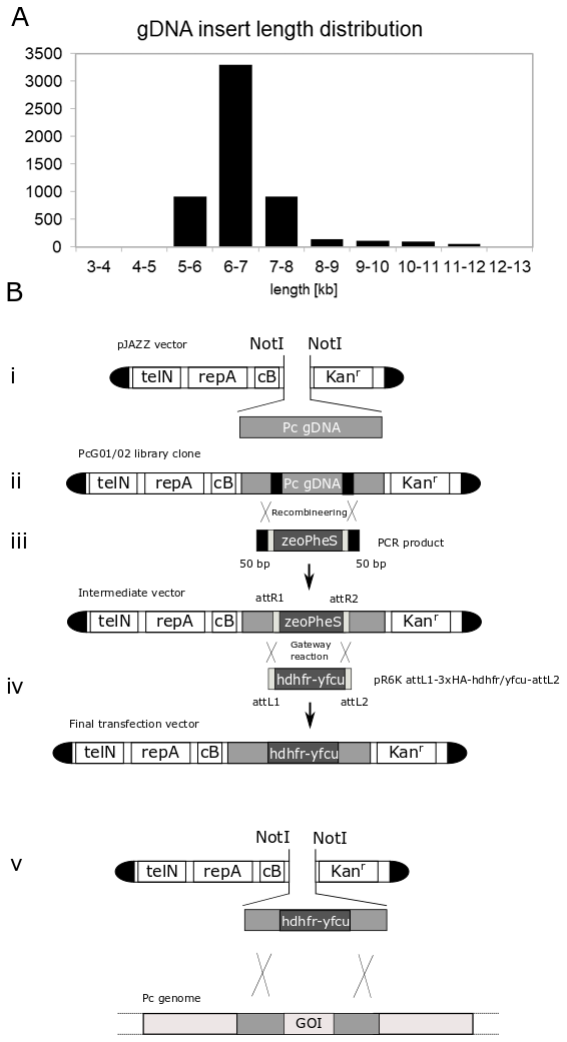
Generation of *Plasmo*GEM resources for genetic modification of *P. chabaudi.* **A**) Insert-size distribution for P. chabaudi genomic DNA (gDNA) clone library showing number of clones for each genomic insert-size category, with an average genomic clone size of 6–7 kb. **B**) The pJAZZ OK Blunt vector ( **i**) was used to clone size-selected P. chabaudi genomic DNA fragments to generate the PcG01 and PcG02 genomic library clones ( **ii**). The pJAZZ-OK Blunt vector encode hairpin telomers (shown in black), a telomerase gene (TelN), replication factor and origin (repA), regulator of replication (cB) and a kanamycin resistance gene (kanR). Genomic library clones are converted into gene targeting vectors by a two-step process. Firstly, recombineering with a PCR amplicon carrying the dual bacterial selectable marker zeocin-pheS (zeo-pheS) flanked by 50 bp homologous targeting sequences (black box) introduces the intended sequence edit ( **iii**). Subsequently, LR-Gateway cloning between AttR and AttL sites (grey box) with the pR6K attL1-3xHA-hdhfr/yfcu-attL2 plasmid, facilitates the exchange of the zeo-pheS cassette for the parasite positive-negative selection marker human dihydrofolate reductase-uridyl phosphoribosyl transferase (hdhfr-yfcu), ( **iv**). Linear transfection-ready vectors are prepared for transfection by NotI digest and integrate with high efficiency into the P. chabaudi genome at the gene of interest (GOI) locus ( **v**).

To create PlasmoGEM resources for *P. chabaudi,* we needed to create such an arrayed library from *P. chabaudi* genomic DNA. We created a gDNA library in the pJazz-OK Blunt cloning vector, consisting of 7104 clones, of which 5523 could be mapped to version 3 of the *P. chabaudi* genome ( Otto *et al.*, 2014) with an average insert size of 6.7kb ( Figure 4A). The cloning efficiency (73%) was similar to our previously generated *P. berghei* gDNA library resource ( Pfander *et al.*, 2011). The library covers at least 50% of the coding-sequence of 4167 protein-coding genes (equivalent to a coverage of 80.7% of all predicted genes in the assembled genome); 3392 (65.7% coverage) genes are covered fully. Using our previously described software, we created automated designs for knock-out vectors for 3665 genes (70.9% coverage) and c-terminal tagging vectors for 3141 genes (60.8% coverage), which are available on our website at https://plasmogem.sanger.ac.uk ( Schwach *et al.*, 2015).

Our initial targets for knock-out vectors were genes implicated in signal transduction, and those predicted to encode exported proteins using a number of criteria including existing annotation of gene function, presence of transmembrane domains and ExportPred algorithm scores ( Sargeant *et al.*, 2006). Using our vector production pipeline that employ a sequential combination of recombinase mediated engineering (recombineering) and Gateway ^TM^ technology ( Pfander *et al.,* 2011), ( Figure 4B), we produced a first batch of 406 (7.9% coverage) barcoded single-gene knock-out vectors that passed quality control by full-length sequencing The vectors are available to request as part of the *Plasmo*GEM project.

### High efficiency transformation of wild-type and PcAS-GFP _ML_ *P. chabaudi* using *Plasmo*GEM vectors

**Figure 5.**
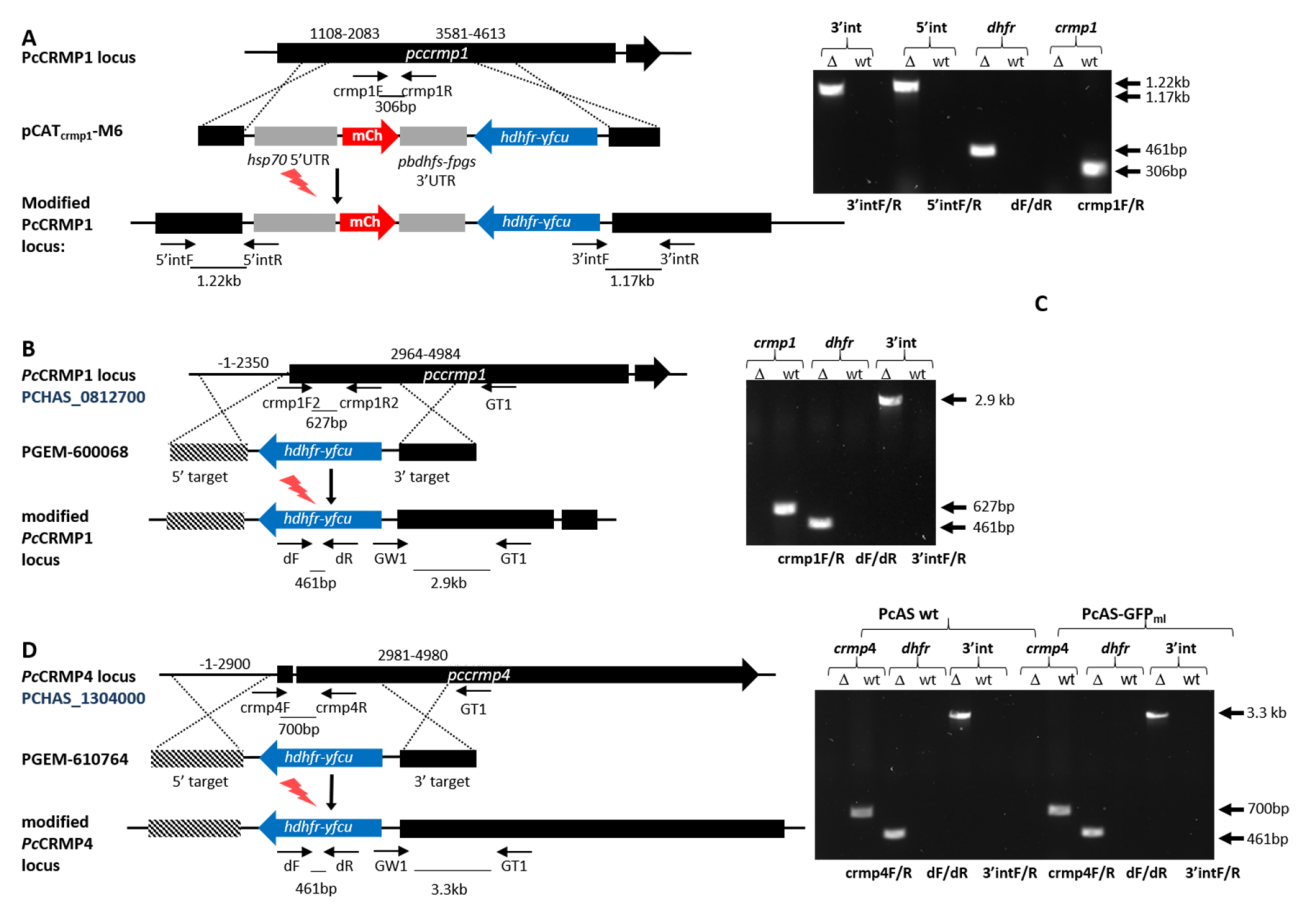
Modification of non-essential loci with pCAT and pJazz plasmids. **A) Left hand panel (LHP)**; schematic representation of the pCAT _crmp1_-M6 plasmid and the *pccrmp1* locus after transfection. **Right hand panel (RHP)**; to verify correct integration into the *pccrmp1* locus, 1.22kb and 1.17kb fragments were amplified from the 5’ and 3’ integration sites using primers 5’intF.crmp1 x 5’intR.crmp1 and 3’intF.crmp1 x 3’intR.crmp1, respectively. To verify deletion of *pccrmp1*, primers crmp1F x crmp1R were used to amplify a 306bp fragment in wild-type but not PcAS-mCh.Δcrmp1 DNA. To verify the presence of *hdhfr*, primers dF1 x dF2 were used to amplify a 461bp fragment in PcAS-mCh.Δcrmp1 but not in wt DNA. **B) LHP**; schematic representation of the PGEM-600068 construct and the *pccrmp1* locus after transfection. **RHP**; to verify correct integration into the *pccrmp1* locus, a 2.9kb fragment was amplified from the 3’ integration site using primers GW1 x GT1.crmp1. To verify deletion of *pccrmp1*, primers crmp1F2 x crmp1R2 were used to amplify a 627bp fragment in wt but not PcAS.Δcrmp1 DNA. To verify the presence of *hdhfr*, primers dF1 x dF2 were used to amplify a 461bp fragment in PcAS.Δcrmp1 but not in wt DNA. **C) Day of patency following transfecti**o **n with pCAT plasmids (n=9) and pJazz constructs (n=10) targeting non-essential loci.** Parasitaemia was monitored by giemsa stain from days 7–16 following transfection. Patency was determined when parasitaemia was at least 0.005%. **D) LHP**; schematic representation of the PGEM-610764 construct and the *pccrmp4* locus after transfection into *P. chabaudi* AS wild-type and PcAS-GFP _ml_ parasites. **RHP**; to verify correct integration into the *pccrmp4* locus, a 3.3kb fragment was amplified from the 3’ integration site using primers GW1 x GT1.crmp4. To verify deletion of *pccrmp4* in wild-type and PcAS-GFP _ml_parasites, primers crmp4F x crmp4R were used to amplify a 700bp fragment in wild-type but not PcAS.Δcrmp1 DNA. To verify the presence of *hdhfr*, primers dF1 x dF2 were used to amplify a 461bp fragment in PcAS.Δcrmp4 but not in wild-type DNA.

To compare the efficiency of transfection between modified pCAT plasmids and pJAZZ-derived *Plasmo*GEM long homology-arm gene disruption vectors, we targeted loci encoding p230p and genes encoding members of the *P. chabaudi* Cysteine Repeat Modular Protein (PcCRMP) family, which are not essential for blood-stage replication ( Thompson *et al.*, 2007), in wild-type *P. chabaudi* AS. As an example, the strategy used to modify the *PCHAS_081270* locus, encoding PcCRMP1, is shown in Figure 5. Patent parasitaemia was observed in pJAZZ vector-transfected lines on day 8–9 following infection and in pCAT vector-transfected lines on day 10–14 ( Figure 5C), suggesting that pJAZZ vectors confer > 64-fold higher transfection efficiency (assuming *P. chabaudi* AS has an 8-fold proliferation rate/cycle).

To next compare the transfection efficiency of PGEM vectors in wild-type and PcAS-GFP _ML_parasites, we transfected a construct targeting PcCRMP4 (PCHAS_1304000; Figure 5D). PcCRMP4-deletant parasites were patent by day 9 post-transfection in both parasite lines and showed integration into the *pcccrmp4* locus and deletion of the gene. Thus, we have demonstrated the generation of marker-free fluorescent mother-lines that can now be genetically-modified using the high-efficiency vectors, available from *Plasmo*GEM, This enhanced toolbox is now ready to kick-start *Plasmodium chabaudi* genetic studies

## Summary

The improvements in the efficiency, practicability and accessibility of *P. chabaudi* transfection techniques reported here have enabled us to reproducibly generate a series of fluorescently-tagged and gene-deletion parasite lines. Transfections were carried out using transfection vectors derived from the versatile pBAT series of plasmids ( Kooij *et al.*, 2012) that can rapidly be adapted for use in gene targeting and tagging strategies, and we have used these to generate fluorescent lines of distinct *P. chabaudi* genotypes (unpublished report). These plasmids also allow recycling of the positive drug-selectable marker and, therefore, sequential genetic modifications. For *P. chabaudi,* this feature may be particularly useful for analysis of the function of multigene families that are not individually essential for blood-stage replication but have been proposed to play a role in immune evasion and modulation, including members of the Plasmodium Cysteine Repeat Modular Protein (PCRMP) family ( Douradinha *et al.*, 2011; Thompson *et al.*, 2007). Thus, we can now study the function of the multi-gene families during both acute infection and chronicity in the face of the host immune response, and we can explore whether family members have overlapping and compensatory roles by performing double and multiple gene deletions, making use of our ability to recycle the selection marker using negative selection.

We have generated a genome-scale *P. chabaudi* genomic library that can effectively be converted into *Plasmo*GEM linear, long homology arm gene targeting vectors that integrate into the *P. chabaudi* genome with high transfection efficiency. This *Plasmo*GEM *P. chabaudi* vector resource can now be used together with fluorescent mother-lines to perform high throughput pooled genetic screens ( Gomes *et al.*, 2015); for example, to identify parasite genes essential for sexual commitment and transmission during chronic infection. Thus, combining the optimisation of transfection technologies with Cas9-based approaches, and the huge vector resource available through PlasmoGEM will create a formidable system. We therefore expect that these improvements in genetic modification of *P. chabaudi* will encourage the research community to adopt this model to answer new research questions that cannot be addressed effectively in other mouse models of malaria.

## Data availability

### Underlying data

PlasmoGem vector sequence and design data are available as a public resource at https://plasmogem.sanger.ac.uk.

Open Science Framework: Marr *et al.*, An enhanced toolkit for the generation of knockout and marker-free fluorescent Plasmodium chabaudi. https://doi.org/10.17605/OSF.IO/65RW3

This project contains the following underlying data:

- Original gel and microscopy images for Figure 1, Figure 2 and Figure 5 in TIF/TIFF format
- pCAT plasmid sequences in DOCX format
- Raw parasitaemia, weight change and anaemia data underlying Figure 3 in XLSX format
- Raw data underlying Figure 5C in XLSX format (Tfn_pCATvpJAZZ)
- Gating strategy for peripheral parasitemia in PPTX format

Data are available under the terms of the Creative Commons Attribution 4.0 International license (CC-BY 4.0).
