# Supplementary material for: An enhanced toolkit for the generation of knockout and marker-free fluorescent Plasmodium chabaudi
Source: Wellcome Open Res. 2020 Jun 24;5:71. Originally published 2020 Apr 17. [Version 2] doi: 10.12688/wellcomeopenres.15587.2 (PMC7236590; doi:10.12688/wellcomeopenres.15587.2)
Supplement: Peer review history

# Peer review: Reviewer response for version 1

**DOI:** 10.21956/wellcomeopenres.17069.r38489

**Published:** 2020-05-18

**Authors:** Robert W. Moon

**Affiliations:** 1 Department of Infection Biology, Faculty of Infectious Diseases, London School of Hygiene and Tropical Medicine, London, UK

Malaria in humans is caused by more than 7 different species of protozoan parasite within the *Plasmodium *genus, with *Plasmodium falciparum* causing the majority of disease and death. Studies into the biology of malaria parasites primarily rely on the use of a few species of human malaria parasites that can be cultured in vitro and 3 different species of rodent malaria parasites which can be maintained in mice in the lab. The functions of a majority of proteins are conserved across all species, so the use of multiple species allows us to make use of key experimental advantages of each system to uncover shared biology. It also offers the opportunity to compare and contrast results to study key differences between the species. Whilst the transfection technology for the rodent malaria parasite *P. chabaudi* has lagged behind other species, it none the less has proved invaluable in the study of key aspects of host-parasite interactions. This work addresses a key current drawback in the *P. chabaudi* model with the presentation of  3 significant advancements in experimental genetics approaches – optimisation of transfection efficiency, generation of marker-less transgenic lines and generation, and testing of a *P. chabaudi* plasmoGEM construct library. The work provides a major technical advancement for the model and provides an important new resource for the field.

 The comments are all minor but I hope should provide some small improvements to the paper.   

**General comments:**

- “two mice to provide infected blood for transfection and eight mice as recipients of parasites transfected with four plasmids in replicate” Why were two mice used for each plasmid per transfection? For replicates, it would be more appropriate to carry these out across different parasite preparations/transfections which are likely the biggest variable for transfection efficiency.
- “a custom software pipeline as previously described for generating a *P. berghei* resource” Whilst the reference describes general parameters an additional direct link to the software and source code (journal requirement) should be added. The version number of software used should also be made clear, I imagine it may be different between the two papers.
- Images in fig1 (smears) are very low quality. I don’t dispute the interpretation but it is very difficult to see the condition of the parasites in that resolution.
- “we tested whether the reversible inhibitor of the *Plasmodium* PKG - C2 ( Collins *et al.*, 2013)” is this the right reference? C2 is not used for synchronisation in this paper. Perhaps it should be https://doi.org/10.1371/journal.ppat.1003344 ^1^ (which has the same first author and year).
- C2 has been used in *P. knowlesii *for transfections for similar reasons. Here viable was found to decrease if held beyond 3 hours. Could the authors comment on the incubation time used (4.5 h) and whether loss of viability was detected after longer incubations? This will help guide correct usage by other labs.
- “undergo schizogony and re-invasion of erythrocytes in static culture ( Figure 2A)” to what extent can *P. chabaudi *be cultured longer term? Can you provide an indicator as to relative growth rate *in vitro*, or whether it can be maintained for multiple cycles? If this has been demonstrated previously could this reference be added?
- Figure 2 title seems to relate to just panel A, the title could be changed to include a reference to transmission states and A) moved to the second sentence. Scale bars are only indicated in one image each for A/B i, ii, iii, but the images are at different magnification. The sporozoite images appear to be at a much greater magnification so need a separate scale bar. The data from Figure 3 are also duplicated in the figure image.
- “terminal tagging vectors for 3141 genes (60.8% coverage), which are available on our website at https://plasmogem.sanger.ac” hyperlink truncated.
- Summary - I would additionally suggest some discussion of where these technologies fit into the wider scheme of those used in other systems. Combining the optimisation of transfection with Cas9 based approaches, and the huge vector resource available through PlasmoGEM could create a formidable system. Also highlight one of the current downsides of the PlasmoGEM system which is that the markers cannot be recycled.
- Summary - *In vitro *reinvasion is mentioned, could short term culture be used for transfection recovery to reduce animal use?

Is the rationale for developing the new method (or application) clearly explained?

Yes

Is the description of the method technically sound?

Yes

Are the conclusions about the method and its performance adequately supported by the findings presented in the article?

Yes

If any results are presented, are all the source data underlying the results available to ensure full reproducibility?

Yes

Are sufficient details provided to allow replication of the method development and its use by others?

Yes

Reviewer Expertise:

Malaria parasite experimental genetics (P. berghei, P. falciparum and P. knowlesi), malaria parasite biology

I confirm that I have read this submission and believe that I have an appropriate level of expertise to confirm that it is of an acceptable scientific standard.

## References

[1] : Malaria parasite cGMP-dependent protein kinase regulates blood stage merozoite secretory organelle discharge and egress. *PLoS Pathog*.2013;9(5) : 10.1371/journal.ppat.1003344 e1003344 10.1371/journal.ppat.1003344 23675297PMC3649973

# Response

**Published:** 2020-06-08

**Authors:** joanne Thompson

**Affiliations:** University of Edinburgh, UK

Thank you for your fast review and for your interesting and insightful comments. We have made the minor textual revisions you have suggested in version 2 of the manuscript and have responded to your specific points inline below; 

- two mice to provide infected blood for transfection and eight mice as recipients of parasites transfected with four plasmids in replicate” Why were two mice used for each plasmid per transfection? For replicates, it would be more appropriate to carry these out across different parasite preparations/transfections which are likely the biggest variable for transfection efficiency.

*Response: Two mice were infected with replicate transfection reactions for each plasmid to ensure that replicates of the modified lines generated originated from independently-transfected merozoites (rather than through amplification of a single transfectant).  We have now up-dated Fig 5C to show that a sufficient number of transfection experiments were carried out, using different parasite preparations, to allow us to demonstrate that transfection efficiency is reproducible across different transfections using different constructs, and is significantly higher using pJAZZ than pCAT plasmids.*

- a custom software pipeline as previously described for generating a *P. berghei* resource” Whilst the reference describes general parameters an additional direct link to the software and source code (journal requirement) should be added. The version number of software used should also be made clear, I imagine it may be different between the two papers.

*Response: The vector designs are not generated by a single piece of software, instead a collection of scripts to automate different tasks. These would require substantial effort before they could be distributed for use by others. However, the rules for vector designs were as previously described and the output of the software pipeline are the gene modification designs, which are fully disclosed and available for scrutiny at https://plasmogem.sanger.ac.uk/ by reviewers and readers. Furthermore, the software pipeline is not essential in the design of gene modification vectors. We could have designed the same constructs manually, using similar design guidelines as used by the software.*

- Images in fig1 (smears) are very low quality. I don’t dispute the interpretation but it is very difficult to see the condition of the parasites in that resolution.

*Response: These images are selected to show that segmented schizonts, containing up to 8 merozoites form in culture.  Images of the whole field of view can be seen in the original data file available at https://doi.org/10.17605/OSF.IO/65RW3.*

- C2 has been used in *P. knowlesii *for transfections for similar reasons. Here viable was found to decrease if held beyond 3 hours. Could the authors comment on the incubation time used (4.5 h) and whether loss of viability was detected after longer incubations? This will help guide correct usage by other labs.

*Response: The parasites are actually cultured in the presence of C2 for 2.5hrs; C2 is added to the culture, together with fresh media, 2 hrs after the culture is set up (see methods).  David Baker had advised that there might be costs to viability with long-term culture, although I should note that the aim here is to synchronise developing schizonts, rather than to arrest schizonts for any period of time. We haven’t tested longer incubation times, so I’m afraid we cannot comment further on this.*

- “undergo schizogony and re-invasion of erythrocytes in static culture ( Figure 2A)” to what extent can *P. chabaudi *be cultured longer term? Can you provide an indicator as to relative growth rate *in vitro*, or whether it can be maintained for multiple cycles? If this has been demonstrated previously could this reference be added?

*Response: It has not been our aim to develop a long-term culture system for P. chabaudi so we cannot comment formally on the relative growth-rate in culture or maintenance for multiple cycles.  On the small number of occasions that we have cultured schizonts with fresh erythrocytes, we have observed re-invasion and development to the young trophozoite stage, but we have not attempted to optimise this procedure or to ‘culture-adapt’ parasite lines.*

- Summary - I would additionally suggest some discussion of where these technologies fit into the wider scheme of those used in other systems. Combining the optimisation of transfection with Cas9 based approaches, and the huge vector resource available through PlasmoGEM could create a formidable system. Also highlight one of the current downsides of the PlasmoGEM system which is that the markers cannot be recycled.

*Response: The hdhfr-yfcu marker used in P. berghei vector resource and the P. chabaudi vectors is recyclable since the hdfr-yfcu cassette is flanked by duplicated pbdhfr 3’utr sequences, which facilitates efficient recombination under 5-FC pressure. It is referenced in the original methods publication by Pfander et al 2011 (Supplementary Figure 1. Map of plasmid pR6K attL1-3xHA-hdhfr-yfcu-attL2).*

- Summary - *In vitro *reinvasion is mentioned, could short term culture be used for transfection recovery to reduce animal use? 

*As described above, we have not optimised techniques for long-term in-vitro culture of P. chabaudi parasites, so cannot perform drug-selection and amplification of the parasites without growth in vivo.*

# Peer review: Reviewer response for version 1

**DOI:** 10.21956/wellcomeopenres.17069.r38487

**Published:** 2020-04-28

**Authors:** Tania F. de Koning-Ward

**Affiliations:** 1 School of Medicine, Deakin University, Waurn Ponds, VIC, Australia

This paper describes the generation of a new fluorescent *P. chabaudi *mother line that can be used to knockout and tag genes using the *P. chabaudi *specific PlasmoGEM vectors that have also been created as part of this study. Together this line and the PlasmoGEM vectors will facilitate the study of gene function in *P. chabaudi, *a rodent species that causes a chronic infection with periodic rescrudescence as a result of its antigenic variation. Hence the ability to genetically manipulate this rodent malaria species will be of value to malaria researchers.

 The methodologies were thoroughly described, the results are robust and conclusions valid. My comments really relate to improving the manuscript for clarity. 

1. I found the methodology described in the second paragraph of the 'Design and generation of PlasmoGEM gene targeting vectors for P. chabaudi' somewhat confusing and whilst this is schematically presented in Figure 4 it would be good to label (i), (ii), etc  and for example indicate which is the pR6K attL1-3xHA-hdhfr/yfcu-attL2 plasmid so that the steps described in the methods can be readily followed in the figure. Also for this figure legend, reword so that mention of (i) comes before (ii). Also show where NotI cuts in the figure.
2. PCR step in 'Transfection and selection of fluorescent P. chabaudi parasites' - it looks like the annealing step has been omitted.
3. Figure 1: include the primers used for the PCRs underneath the figure for greater clarity. E.g. presumably in Fig 1B df/dr was used in first panel and recF and recR in second?
4. Table 1: no restriction sites have been underlined.
5. Results: Design of transfection vectors: one has to go to the methodologies to see what pCAT230p-G6 and pCAT230p-M6 are. Indicate they are modified versions of the pSIL6 vector that incorporates...
6. Figure 2. Panel 2Ai - it is hard to tell if these are rosettes or if there is just a high density of cells on the slide.
  There is no reference to figure 2ii in the text.
  Figure 2 also contains figure 3 - please delete.
7. Figure 3: Panel B and C are not described in the text at all. How do these compare to blood stage parasites? Best if they are also on the same graph. Please confirm that the mice increase in weight after infection rather than decrease.
8. Figure 5: Again it would be best to show the oligo combinations used in the PCR beneath the figure rather than in the figure legend for greater clarity. Panel C would be better as a scatter plot to show variability - is the difference also significant?
9. Minor errors:
  keywords - Transfection should be lower-case t.
  c-terminal tagging - The c should be in capitals.
  gelectrophoresis In 'Transfection' in methodology, there should be a space between gel and electrophoresis.

Is the rationale for developing the new method (or application) clearly explained?

Yes

Is the description of the method technically sound?

Yes

Are the conclusions about the method and its performance adequately supported by the findings presented in the article?

Yes

If any results are presented, are all the source data underlying the results available to ensure full reproducibility?

Yes

Are sufficient details provided to allow replication of the method development and its use by others?

Yes

Reviewer Expertise:

molecular biology, malaria transfection, animal studies

I confirm that I have read this submission and believe that I have an appropriate level of expertise to confirm that it is of an acceptable scientific standard.

# Response

**Published:** 2020-06-08

**Authors:** joanne Thompson

**Affiliations:** University of Edinburgh, UK

Thank you for your fast response and for the very interesting and constructive comments you have made. We have made the minor changes you have requested in the text and figure annotation in version 2 of the manuscript and have replied to specific points in the text below.

>6. Figure 2. Panel 2Ai - it is hard to tell if these are rosettes or if there is just a high density of cells on the slide. 

*To clarify, the images shown are of live parasites in culture; a comment has been included in the figure legend to explain this. Since they are live, there is a lot of movement of cells in the cultures, and only cells that are tightly adherent remain together.  This is only observed for schizont-infected erythrocytes, and erythrocytes infected with all other stages do not form rosettes (please refer to original data for more images of this, available at https://doi.org/10.17605/OSF.IO/65RW3).  Interestingly, we have observed that the percentage of schizonts forming rosettes varies between P. chabaudi strains and decreases after serial blood-passage.*

>7. How do these compare to blood stage parasites? Best if they are also on the same graph. 

*The kinetics of parasitaemia and infection of wt P. chabaudi AS parasites have been extremely well documented, including in the Spence et al (2013) paper cited here.  These publications have demonstrated that, although there may be some variation (likely caused by a number of factors, including mouse housing conditions and number of times the parasites have been serially blood-passaged since mosquito-transmission), serially blood passaged P. chabaudi parasites reach a peak parasitaemia of ~20% between days 7-9 of infection, and are then resolved, whilst recently mosquito-transmitted parasitaemia are attenuated, reaching a peak parasitaemia of <5%.  Since we have demonstrated that the PcAS-GFP _ml _parasites used in this study show the same parasitaemia kinetics and virulence, we did not believe that it was justified to increase our animal usage by carrying out concurrent wt infections.  We have therefore described the wt infection dynamics in the text and cited the relevant previous reports.*
